# SARS-CoV-2 Infection Is Associated with an Accelerated eGFR Decline in Kidney Transplant Recipients up to Four Years Post Infection

**DOI:** 10.3390/diagnostics15091091

**Published:** 2025-04-25

**Authors:** Shawn Qiu, Roham Hadidchi, Aditi Vichare, Justin Y. Lu, Wei Hou, Sonya Henry, Enver Akalin, Tim Q. Duong

**Affiliations:** 1 Department of Radiology, Albert Einstein College of Medicine and Montefiore Medical Center, Bronx, NY 10461, USA; shawn.qiu@einsteinmed.edu (S.Q.); roham.hadidchi@einsteinmed.edu (R.H.); aditi.vichare@einsteinmed.edu (A.V.); justin.lu@einsteinmed.edu (J.Y.L.); weihouanhui@gmail.com (W.H.); sonya.henry@einsteinmed.edu (S.H.); 2 Department of Medicine (Nephrology), Albert Einstein College of Medicine and Montefiore Medical Center, Bronx, NY 10461, USA; eakalin@montefiore.org

**Keywords:** long-COVID, solid organ transplant, SARS-CoV-2, estimated glomerular filtration rate

## Abstract

**Background/Objectives:** Although kidney transplant recipients (KTRs) who are immune-compromised have been shown to be at high risk of adverse acute COVID-19 outcomes (i.e., mortality and critical illness), the long-term outcomes of KTRs with a history of SARS-CoV-2 infection are unknown. We aimed to compare long-term outcomes of KTRs with and without exposure to SARS-CoV-2. **Methods:** This study retrospectively evaluated 1815 KTRs in the Montefiore Health System from 4 January 2001 to 31 January 2024. The final cohorts consisted of KTRs who survived COVID-19 (*n* = 510) and matched KTRs without COVID-19 (*n* = 510, controls). Outcomes were defined as all-cause mortality and changes in estimated glomerular filtration rate (eGFR) and urine protein to creatinine ratio (UPCR) from 30 days up to four years post index date. Kaplan–Meier survival analysis and Cox proportional modeling were performed for mortality. Generalized estimating equations were used to analyze changes in eGFR and UPCR across time. **Results:** There was no significant group difference in long-term all-cause mortality (adjusted hazard ratio = 0.66, [0.43, 1.01] *p* = 0.057). eGFR in controls and COVID-19 patients before infection similarly decreased −0.98 units/year [−1.50, −0.46]. By contrast, eGFR declined at a significantly greater rate (−1.80 units/year [−2.45, −1.15]) in KTRs after COVID-19 compared to KTRs without COVID-19. This association was only seen among male and not female KTRs. COVID-19 status was not significantly associated with rate of change in UPCR or acute kidney rejection rate. **Conclusions:** SARS-CoV-2 infection was associated with an accelerated decline in eGFR up to four years post infection, suggesting potential long-term implications for graft health. These findings underscore the importance of vigilant monitoring and management of kidney function post SARS-CoV-2 infection in this vulnerable population.

## 1. Introduction

SARS-CoV-2 infection has disproportionately affected individuals with a compromised immune system [1,2]. Among these, kidney transplant recipients (KTR) represent an especially vulnerable group [3,4,5]. Severe COVID-19 triggers pathological hyperinflammation and dysregulated host-immune responses, which could lead to multi-organ failures [6,7,8]. This inflammatory milieu could be harmful to graft organs of KTRs who are already immune-compromised. Furthermore, immune suppression in KTRs may impair the ability to mount an effective immune response against SARS-CoV-2, exacerbating COVID-19 disease severity and prolonging recovery.

A few studies have documented that KTRs with COVID-19 are at elevated risk of experiencing acute adverse COVID-19 outcomes (i.e., mortality or critical illness) compared to COVID-19 patients without kidney transplantation or KTRs without COVID-19 [9,10,11,12,13]. There have been no studies to date that have investigated the long-term outcomes comparing KTRs with and without a history of SARS-CoV-2 infection. It is possible that COVID-19 could continue to exert lasting effects in KTRs, given their heightened vulnerability, long after acute COVID-19 symptoms have resolved. Specifically, a history of COVID-19 could result in accelerated kidney function decline and increased graft rejection and mortality post infection.

We thus investigated the long-term outcomes of KTRs with and without SARS-CoV-2 infection up to four years post infection. Outcomes included all-cause mortality, graft rejection, and changes in estimated glomerular filtration rate (eGFR) and urine protein to creatinine ratio (UPCR). Comparisons were made between KTRs with and without SARS-CoV-2 infection. Our data came from the Montefiore Health System, which serves a large, diverse urban population in the Bronx, an epicenter of the early COVID-19 pandemic and subsequent surges of infection

## 2. Materials and Methods

### 2.1. Data Sources

This retrospective single-health system study was approved by the Einstein-Montefiore Institutional Review Board with an exemption for informed consent (#2021-13658). Data were obtained from the Montefiore Health System, which consists of multiple hospitals in the Bronx, the lower Hudson Valley, and Westchester County. Data were extracted as described previously [14,15,16,17,18,19,20,21].

Data extraction queried records for kidney transplants from 4 January 2001 to 31 January 2024 and SARS-CoV-2 polymerase chain reaction (PCR) tests from 1 February 2020 to 31 January 2024. There were 1815 patients with a history of kidney transplant during this time frame. Individuals who had a positive SARS-CoV-2 PCR test were placed in the COVID-19 group, and all others without a documented positive PCR test were placed in the non-COVID (control) group. Index date was defined as the date of positive PCR test for the COVID-19 cohort. All visits occurring after 1 March 2020 were identified for the control patients, who were 1:1-matched to the COVID-19 patients based on observation time. For each COVID-19 patient, a non-COVID patient was identified, whose post-1 March 2020 visit yielded a similar follow-up period (within one month). This approach was designed to ensure comparability in observation time between groups.

### 2.2. Data Collection

Patient demographics and comorbidities were extracted. Demographic data consisted of age at index date, sex, ethnicity, and race. Pre-existing comorbidities at index date included type 2 diabetes (T2DM), hypertension (HTN), asthma, chronic obstructive pulmonary disease (COPD), and cardiovascular diseases (CVDs, a composite of chronic heart failure, coronary artery disease, and history of myocardial infarction). Diagnosis of polycystic kidney disease or glomerulonephritis before transplant date was also collected. eGFR and UPCR at baseline (within the two years prior to the index date) and at latest follow-up visit were also collected and displayed in units of mL/min/body surface area (BSA) and mg/g, respectively. Essentially all patients had longitudinal eGFR measurements available for analysis (at least one pre- and one post-index date, 99.02% of the COVID-19 group and 96.47% of the non-COVID group), with each patient having an average of 73 recorded measurements. Most patients had longitudinal UPCR measurements available for analysis (92.16% of the COVID-19 group and 90.78% of the non-COVID group), with each patient having an average of 10 measurements. Graft biopsies and biopsy-confirmed kidney rejections were also collected.

### 2.3. Outcomes

Outcomes included all-cause mortality and biopsy-confirmed graft rejection 30 days to four years post index date, and changes in eGFR and UPCR starting two years before index date to latest follow-up.

### 2.4. Statistical Analysis

Python version 3.10.12 (Python Software Foundation, Wilmington, DE, USA) and the lifelines package, the survival, survminer, and cmprsk packages in RStudio version 4.3.2 (RStudio, PBC, Boston, MA, USA), and GraphPad Prism 9 version 10.1.1 (GraphPad Software, Boston, MA, USA) were used for data processing and statistical analyses. Group comparison of categorical variables used the chi-square test, and group comparison of continuous variables used the independent *t*-test. Kaplan–Meier curves, log-rank, and Cox proportional hazards analyses were performed for all-cause mortality. For eGFR and UPCR analyses, generalized estimating regression equations with an exchangeable correlation structure were used to account for repeated measures within individuals, starting two years prior to index date and up to latest follow-up visit. Time (in years from transplant date) was modeled as a continuous variable, and both time and group (COVID vs. non-COVID) were included as main effects. Interaction terms between time and COVID-19 status were used to estimate differences in trajectories over time. UPCR values had to be log-transformed prior to modeling to achieve normal distribution, and coefficients were exponentiated for easier interpretability [22]. *p*-values less than 0.05 were considered statistically significant.

## 3. Results

### 3.1. Cohort Selection and Demographics

**Figure 1:**
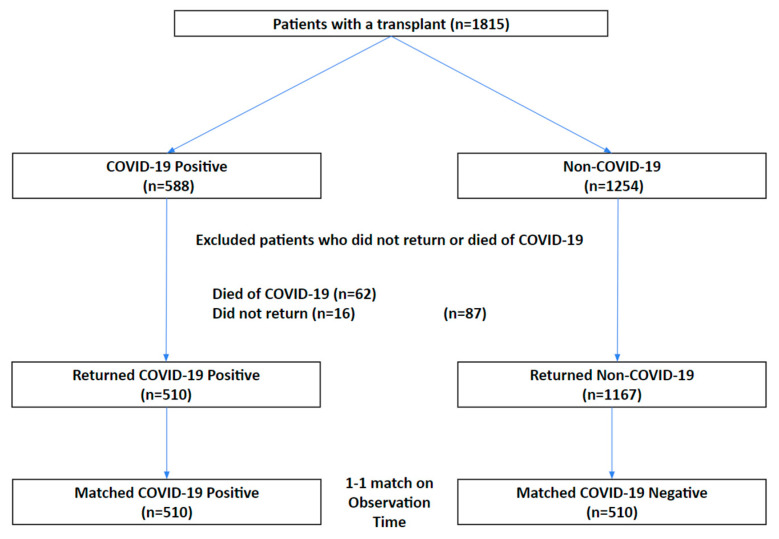
Patient selection flowchart.

Figure 1 shows the patient selection flowchart. There were 1815 patients with a history of kidney transplantation, of which 588 had a positive COVID-19 PCR test and 1254 did not (controls). Acute COVID-19 mortality was 10.54% (62/588) within the first 30 days of COVID-19 positive test. There were 510 COVID-19 patients and 1167 controls who returned to our health system 30 days post-index date or later. A 1:1 match by observation time was performed to obtain 510 patients for the control group.

Table 1 summarizes the baseline demographics, comorbidities, baseline eGFR, and UPCR of the two cohorts. There were no group differences in sex, age, observation time, race, and ethnicity (*p* > 0.05). On the index date, COVID-19 patients had received their transplant more recently than non-COVID patients (4.61 vs. 6.48 years (median), *p* < 0.001). The COVID-19 cohort had a higher prevalence of T2DM (71.76% vs. 60.59%, *p* = 0.045), asthma (20.59% vs. 12.75%, *p* = 0.004), and CVD (65.88% vs. 51.76%, *p* = 0.009) compared to controls. Most (83.68%) COVID-19 patients were hospitalized during the acute phase of the infection, with 15 experiencing critical illness (intensive care unit admission or invasive mechanical ventilation use). Some 190 (37.25%) of COVID-19 patients were given Remdesivir during acute infection. The COVID-19 cohort had a lower baseline eGFR (46 vs. 48 mL/min/BSA (median), *p* < 0.001) but a similar UPCR (209 vs. 242 mg/g (median), *p* = 0.14) compared to the non-COVID cohort.

### 3.2. Clinical Outcomes

**Figure 2:**
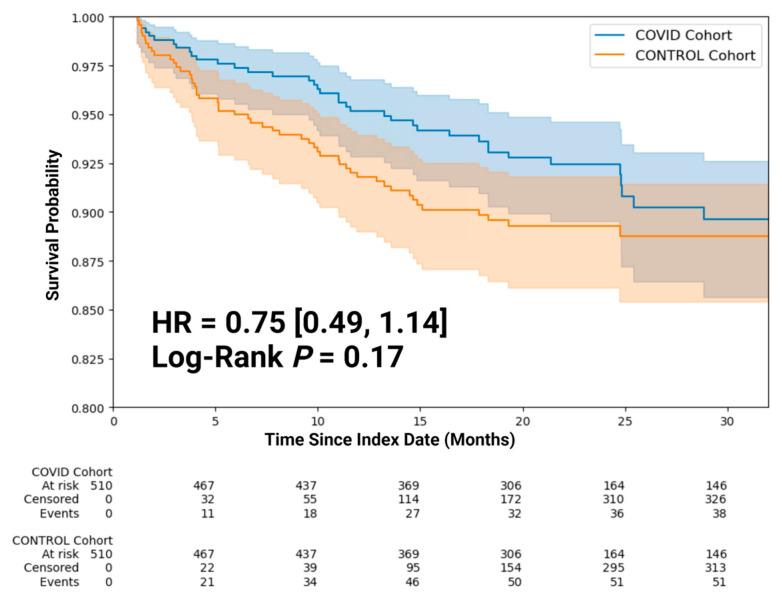
Kaplan–Meier curve of post-index date (all-cause mortality).

Figure 2 shows the Kaplan–Meier survival curve of KTRs with and without COVID-19. Log-rank analysis showed that risk of all-cause mortality (HR = 0.75 [0.49, 1.14], *p* = 0.17) was similar between the COVID-19 and control groups.

Table 2 shows the adjusted hazard ratios (aHRs) for all-cause mortality. COVID-19 status was not a significant predictor of all-cause mortality (aHR = 0.66 [0.43, 1.01], *p* = 0.057). Older age at index date (aHR = 1.05 [1.03, 1.07], *p* < 0.001) and lower baseline eGFR (aHR = 0.98 [0.98, 0.99], *p* < 0.001) were significantly associated with greater risk of all-cause mortality.

There were 83 (16.27%) KTRs with kidney biopsies in the COVID-19 cohort and 48 (9.41%) in the control cohort, which confirmed post-index date kidney rejection in one COVID-19 and two control patients.

### 3.3. Biomarker Outcomes

**Figure 3:**
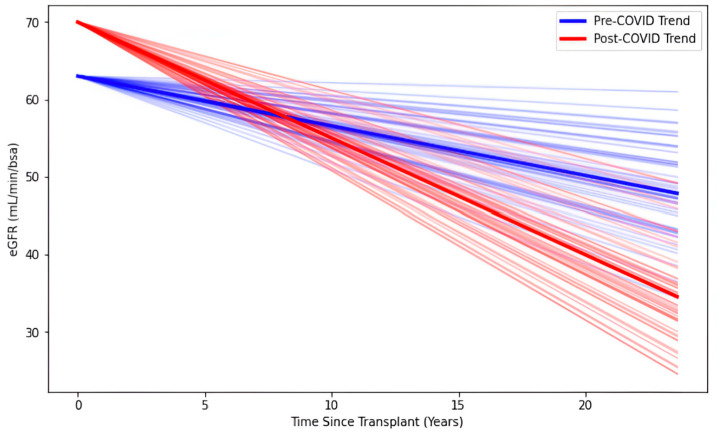
Graphical representation of results from the generalized estimating equation regression model tracking changes in estimated glomerular filtration rate (eGFR).

To investigate the potential association of COVID-19 with eGFR trajectory, a generalized estimating equations model was used, tracking changes in eGFR starting two years before the index date and up to the latest follow-up visit. eGFR declined by −0.98 units/year [−1.50, −0.46] among controls and COVID-19 patients before infection. After SARS-CoV-2 infection, the rate of eGFR decline was accelerated to −1.80 units/year [−2.45, −1.15]. These results are graphically shown in Figure 3. When analysis was stratified by sex, this association of COVID-19 with declining eGFR only held among males and not females (Supplementary Table S1). The full results of other variables from the model are shown in Table 3. The estimated eGFR at the time of transplantation for controls was 63.70 units; males were 3.74 units higher, and those with pre-existing CVD were 11.90 units lower.

A similar longitudinal analysis showed that UPCR increased by an estimated 5% per year for all patients, but there was no association with COVID-19 status, and those with CVD had an estimated 16% greater UPCR at the time of transplant (Table 4).

To evaluate potential sex differences in eGFR and UPCR outcomes, a sex-stratified analysis was performed in Supplementary Table S1. Among males, those with COVID-19 had a faster decline in eGFR over time (−1.12 [−1.65, −0.59] units/year). This association was not seen in females. COVID-19 was also not associated with changes in UPCR among male or female KTRs.

## 4. Discussion

This study investigated the clinical outcomes of KTRs with and without COVID-19 up to four years post COVID-19 in a large diverse population in the Bronx, New York. COVID-19 status in KTRs was not associated with increased risk of all-cause mortality or changes in UPCR but was linked with an accelerated decline in eGFR, particularly in male patients.

KTRs with and without a history of SAR-CoV-2 infection had similar demographics. Although KTRs in both groups had a high prevalence of pre-existing conditions, KTRs with COVID-19 had a higher prevalence of T2DM and CVD compared to KTRs without COVID-19. Notably, 83.68% of COVID-19 patients were hospitalized for acute infection, suggesting that admission may have been a precautionary measure for many of these immunosuppressed patients. Similar COVID-19 hospitalization rates (86.5%) have been observed among liver transplant patients [23].

The COVID-19 group consisted of more recent transplant recipients than the control group by approximately two years on average. It is possible that the elevated level of immunosuppression in recent KTRs increased susceptibility to infection. It is also possible that the elevated level of immunosuppression in recent KTRs contributed to worse long-term outcomes. However, multivariate analysis showed that recency of transplant (time from transplant to index date) was not significantly associated with all-cause mortality or eGFR and UPCR, as the generalized estimating modeling equations of eGFR and UPCR treated transplant date as time zero.

Although COVID-19 status was not significantly associated with all-cause mortality after adjusting for confounders, KTRs with COVID-19 showed a slightly more favorable mortality outcome which could be due to survivor effects (i.e., patients who survived acute COVID-19 illness could be healthier). Older age and lower baseline eGFR were significant predictors of increased all-cause mortality risk, as expected, consistent with the existing literature [24,25]. Although there are no studies that directly compare the long-term mortality of KTRs with and without COVID-19, a recent meta-analysis showed that KTRs with SARS-CoV-2 infection were at an elevated risk of acute mortality and acute kidney injury compared to non-transplant COVID-19 patients [26]. We were unable to conclude whether there was an association between COVID-19 status and graft rejection, as graft rejection in both groups was relatively rare. Prior studies have faced similar challenges [27], but those with sufficient sample size have seen no acute [28,29] or long-term [30,31] association between COVID-19 and kidney graft rejection. We found no association between COVID-19 and changes in UPCR across time.

The key finding of the present study is that KTRs who survived SARS-CoV-2 infection experienced a significantly faster decline in eGFR compared to KTRs without SARS-CoV-2 infection up to four years post infection, particularly in males, suggesting long-term implications for graft health. These findings are consistent with broader concerns about the vulnerability of this population to adverse outcomes following SARS-CoV-2 infection. There are no similar studies with which to compare. A related study by Malinowska et al. reported that COVID-19 severity was associated with a greater decline in eGFR among males (but not female) one year post-recovery, with a decrease of 13.94 percentage points (95% CI: −25.13 to −2.76, *p* = 0.015) [32]. In contrast to our study, this study did not compare with KTRs without SARS-CoV-2 infection.

There are multiple interconnected mechanisms that could have contributed to the accelerated decline in eGFR in KTRs with SARS-CoV-2 infection. Immune-mediated injury associated with the immunological dysregulation and hyperinflammatory response seen in acute COVID-19 could have lasting effects [33,34,35,36]. The excessive production of pro-inflammatory cytokines such as IL-6 and TNF-α may also result in chronic low-grade inflammation, fibrosis, and scarring, potentially leading to progressive kidney dysfunction over time [37,38,39,40,41,42,43]. Thromboembolic complications, frequently seen in COVID-19 patients, may also contribute to long-term kidney damage as COVID-19 is known to cause microvascular injury, endothelial dysfunction, and increased coagulation, leading to microthrombi formation [44,45]. Hypoxic injury during the acute phase of COVID-19, particularly in patients with severe respiratory complications, may also play a role [46,47]. Pulmonary and cardiovascular stress during acute COVID-19 could cause hemodynamic disruption. The kidneys are highly sensitive to changes in oxygenation, and episodes of systemic hypoxia could have long-lasting effects on kidney perfusion and function [48,49,50,51]. Hypoxia-induced tubular injury may not fully resolve, potentially contributing to declining eGFR. Finally, KTRs have significant comorbidities which could be exacerbated by COVID-19 illness indirectly, resulting in accelerated eGFR decline [52].

Post-COVID-19 renal impairment is an emerging concern, particularly in KTRs, who are already at increased risk of renal dysfunction due to chronic immunosuppression and pre-existing comorbidities like T2DM and HTN [53,54,55,56]. Acute kidney injury during acute SARS-CoV-2 infection has been frequently reported among KTRs [26]. Our findings span four years, demonstrating that even after recovery from acute infection, long-term declines in renal filtration capacity occur in the following years. Further research is warranted to determine whether specific therapeutic strategies, such as early anti-inflammatory treatment or adjustments in immunosuppression, may mitigate this decline and preserve long-term graft filtration function [26,57].

### Strengths and Limitations

The strengths of this study included a large and diverse cohort of KTRs in an urban population affected by the initial and subsequent surges of infection, long follow-up period, and robust analysis to evaluate rates of eGFR and UPCR changes.

There are several limitations. Data were limited to those who returned to our health system for medical care. Patients who returned could have had more severe COVID-19, but our data also include patients who came back for routine care and checkups. The Montefiore health system is the predominant health provider in the Bronx. Furthermore, we did not analyze data for different strains or vaccination status. Vaccine status, an important variable especially for KTRs [58,59,60,61,62], was not reliably recorded if patients received vaccines outside of the Montefiore Health System. Our observation time was up to four years post infection, but longer follow-up time is needed.

Additionally, the use of eGFR to assess kidney function has limitations as it may be influenced by cardiovascular status, blood pressure variability, and medications, and nonlinear changes with age and disease states. Furthermore, in certain individuals and conditions, eGFR may not correlate well with actual GFR. Although other markers such as urinary collagen fragments (endotrophin, procollagen III, endostatin) could offer additional insights into fibrotic or structural kidney changes, they are not yet standardized for routine clinical use and were not available at our institution during the study period. COVID-19 patients had a higher prevalence of pre-existing comorbidities, which while adjusted for with multivariate regression, does not preclude the possibility of residual confounding. As with any retrospective study, there could be other unintended patient selection biases and latent confounds.

## 5. Conclusions

SARS-CoV-2 infection is associated with an accelerated decline in eGFR up to four years post infection, suggesting significant long-term implications for graft health. These findings underscore the importance of vigilant monitoring of kidney function post-COVID and careful adjustment of immunosuppressive regimens to prevent further damage.

## Figures and Tables

**Table 1:** Characteristics of patients with history of kidney transplant stratified by SARS-CoV-2 exposure status matched for observation time. COPD, chronic obstructive pulmonary disease. eGFR, estimated glomerular filtration rate. UPCR, urine protein to creatinine ratio. SD, standard deviation. IQR, interquartile range. N/A, not applicable.

	COVID+ (*n* = 510)	COVID− (*n* = 510)	*p*-Value
Male, *n* (%)	295 (57.84%)	312 (61.18%)	0.381
Age at Index Date in Years, mean (SD)	59 (24)	59 (23)	0.689
Follow-Up Time in Months, mean (SD)	23 (12)	23 (12)	0.955
Transplant Date to Index Date in Years, median (IQR)	4.61 (1.71, 9.67)	6.48 (3.63, 11.48)	<0.001
**Race and Ethnicity, *n* (%)**			
White	52 (10.2%)	56 (10.98%)	0.710
Black	189 (37.06%)	189 (37.06%)	0.883
Other Race	269 (52.75%)	265 (51.96%)	0.715
Hispanic	212 (41.57%)	191 (37.45%)	0.092
**Pre-Existing Comorbidities, *n* (%)**			
Type 2 Diabetes	366 (71.76%)	309 (60.59%)	0.045
Hypertension	506 (99.22%)	492 (96.47%)	0.675
Asthma	105 (20.59%)	65 (12.75%)	0.004
COPD	62 (12.16%)	41 (8.04%)	0.079
Cardiovascular Diseases	336 (65.88%)	264 (51.76%)	0.009
**Kidney Diseases Prior to Transplant, *n* (%)**			
Polycystic Kidney Disease	23 (4.51%)	15 (2.94%)	0.25
Glomerulonephritis	37 (7.25%)	34 (6.67%)	0.81
**Acute COVID-19 Treatments, *n* (%)**			
Hospitalization Due to COVID-19	432 (83.68%)	N/A	N/A
Critical Illness Due to COVID-19	15 (2.94%)	N/A	N/A
Remdesivir	190 (37.25%)	N/A	N/A
**Baseline Biomarkers Pre-Index Date**			
eGFR (mL/min/BSA), median (IQR)	(*n* = 498) 46 (29, 60)	(*n* = 454) 48 (13, 60)	<0.001
UPCR (mg/g), median (IQR)	(*n* = 440) 209 (117, 453)	(*n* = 431) 242 (131, 500)	0.14

**Table 2:** Cox proportional adjusted model hazard ratios (aHRs) and their 95% confidence intervals (CI) for all-cause mortality. eGFR, estimated glomerular filtration rate. BSA, body surface area.

Covariate	Mortality aHR [95% CI]	*p*-Value
SARS-CoV-2 Infection	0.66 [0.43, 1.01]	0.057
Age at Index Date (Years)	1.05 [1.03, 1.07]	<0.001
Transplant to Index Date (Years)	0.99 [0.96, 1.03]	0.75
eGFR at Baseline (mL/min/BSA)	0.98 [0.98, 0.99]	<0.001
Male vs. Female Sex	1.33 [0.86, 2.06]	0.20
Type 2 Diabetes	1.39 [0.81, 2.40]	0.23
Cardiovascular Diseases	1.54 [0.90, 2.63]	0.11

**Table 3:** Generalized estimating equation regression model tracking changes in estimated glomerular filtration rate (eGFR) (mL/min/body surface area).

Predictor	eGFR β [95% CI]	*p*-Value	Standard Error	*t*-Value
Time Since Transplant (Years)	−0.98 [−1.50, −0.46]	<0.001	0.27	−3.69
Time Since Transplant (Years) × SARS-CoV-2 Infection	−0.82 [−1.19, −0.45]	<0.001	0.19	−4.33
Intercept	63 [57, 70]	<0.001	3.23	19.73
SARS-CoV-2 Infection	7.12 [4.06, 10.18]	<0.001	1.56	4.57
Age (Years)	−0.03 [−0.13, 0.08]	0.637	0.055	−0.47
Male vs. Female Sex	3.84 [0.90, 6.78]	0.011	1.50	2.56
Type 2 Diabetes	−0.22 [−3.63, 3.19]	0.901	1.74	−0.13
Cardiovascular Diseases	−11.90 [−15.2, −8.60]	<0.001	1.69	−7.06

**Table 4:** Generalized estimating equation regression model tracking changes in urine protein to creatinine ratio (UPCR) (mg/g). UPCR had to be log-transformed due to non-normal distribution, which is why the results are presented as exponentiated coefficients (e^β^) for easier interpretability.

Predictor	UPCR e^β^ [95% CI]	*p*-Value	Standard Error	*t*-Value
Time Since Transplant (Years)	1.05 [1.04, 1.07]	<0.001	0.009	6.068
Time Since Transplant (Years) × SARS-CoV-2 Infection	1.01 [0.99, 1.02]	0.45	0.008	0.762
Intercept	121 [93, 158]	<0.001	0.134	35.901
SARS-CoV-2 Infection	1.15 [0.99, 1.33]	0.076	0.077	1.777
Age (Years)	1.00 [1.00, 1.01]	0.11	0.002	1.617
Male vs. Female Sex	1.06 [0.93, 1.21]	0.38	0.068	0.883
Type 2 Diabetes	1.11 [0.96, 1.29]	0.17	0.077	1.374
Cardiovascular Diseases	1.16 [1.00, 1.33]	0.047	0.073	1.982

## Data Availability

The original contributions presented in this study are included in the article/Supplementary Materials. Further inquiries can be directed to the corresponding author.

## Supplementary Materials

The following supporting information can be downloaded at https://www.mdpi.com/article/10.3390/diagnostics15091091/s1, Table S1: Generalized estimating equations regression model tracking changes in (A) estimated glomerular filtration rate (eGFR) (mL/min/body surface area), and (B) urine protein to creatinine ratio (UPCR) (mg/g) among males and females. UPCR had to be log-transformed due to non-normal distribution, which is why results are presented as exponentiated coefficients (e^β^) for easier interpretability.

## Abbreviations

The following abbreviations are used in this manuscript:

KTR	Kidney transplant recipient
eGFR	Estimated glomerular filtration rate
UPCR	Urine protein to creatinine ratio
aHR	Adjusted hazard ratio
PCR	Polymerase chain reaction
T2DM	Type 2 diabetes mellitus
HTN	Hypertension
COPD	Chronic obstructive pulmonary disease
CVD	Cardiovascular disease
