# Supplementary material for: SARS-CoV-2 Infection Is Associated with an Accelerated eGFR Decline in Kidney Transplant Recipients up to Four Years Post Infection
Source: Diagnostics (Basel). 2025 Apr 25;15(9):1091. doi: 10.3390/diagnostics15091091 (PMC12072077; doi:10.3390/diagnostics15091091)
Supplement: Supplementary file 1 [file diagnostics-15-01091-s001.zip › diagnostics-3559659-supplementary.pdf]

Supplementary Table S1. Generalized estimating equations regression model tracking changes in **A)** estimated glomerular filtration rate (eGFR) (mL/min/body surface area), and **B)** urine protein to creatinine ratio (UPCR) (mg/g) among males and females. UPCR had to be log-transformed due to non-normal distribution, which is why results are presented as exponentiated coefficients (e^{β}) for easier interpretability.
SE, standard error.

**A) eGFR**	**Males**				**Females**			
	eGFR β [95% CI]	*p*-value	SE	z-value	eGFR β [95% CI]	*p*-value	SE	t-value
Time Since Transplant (Years)	-1.13 [-1.77, -0.49]	**0.001**	0.33	-3.45	-1.20 [-2.04, -0.35]	**0.0060**	0.43	-2.77
Time Since Transplant × SARS-CoV-2 Infection	-1.12 [-1.65, -0.59]	**<0.001**	0.27	-4.14	-0.07 [-1.35, 1.21]	0.91	0.65	-0.11
Intercept	67.06 [59.23, 74.90]	**<0.001**	4.00	16.78	73.69 [63.74, 83.64]	**<0.001**	5.08	14.52
SARS-CoV-2 Infection	8.55 [4.43, 12.68]	**<0.001**	2.10	4.07	-4.92 [-15.48, 5.64]	0.36	5.39	-0.91
Age (Years)	0.02 [-0.12, 0.15]	0.81	0.070	0.24	-0.07 [-0.18, 0.05]	0.26	0.06	-1.13
Type-2 Diabetes	-1.30 [-5.89, 3.29]	0.58	2.34	-0.56	-0.89 [-4.60, 2.82]	0.64	1.89	-0.47
Cardiovascular Diseases	-12.05 [-16.56, -7.54]	**<0.001**	2.30	-5.24	-10.53 [-14.19, -6.86]	**<0.001**	1.87	-5.63

**B) UPCR**	**Males**				**Females**			
	UPCR e^{β} [95% CI]	*p*-value	SE	z-value	UPCR e^{β} [95% CI]	*p*-value	SE	t-value
Time Since Transplant (Years)	1.05 [1.03, 1.08]	**<0.001**	0.014	3.94	1.05 [1.03, 1.08]	**<0.001**	0.011	4.54
Time Since Transplant × SARS-CoV-2 Infection	1.00 [0.98, 1.02]	0.79	0.011	0.26	1.01 [0.98, 1.03]	0.53	0.012	0.63
Intercept	108.92 [75.05, 157.90]	**<0.001**	0.189	24.81	157.59 [111.01, 220.01]	**<0.001**	0.17	28.97
SARS-CoV-2 Infection	1.22 [1.01, 1.47]	**0.035**	0.094	2.11	1.07 [0.84, 1.37]	0.58	0.13	0.55
Age (Years)	1.00 [0.998, 1.01]	0.20	0.003	1.30	1.00 [0.997, 1.01]	0.38	0.0030	0.88
Type-2 Diabetes	1.08 [0.87, 1.34]	0.50	0.11	0.68	1.09 [0.88, 1.33]	0.43	0.11	0.79
Cardiovascular Diseases	1.38 [1.12, 1.70]	**0.0020**	0.106	3.06	0.98 [0.81, 1.20]	0.86	0.10	-0.17
